# A biofeedback cycling training to improve locomotion: a case series study based on gait pattern classification of 153 chronic stroke patients

**DOI:** 10.1186/1743-0003-8-47

**Published:** 2011-08-24

**Authors:** Simona Ferrante, Emilia Ambrosini, Paola Ravelli, Eleonora Guanziroli, Franco Molteni, Giancarlo Ferrigno, Alessandra Pedrocchi

**Affiliations:** 1 NearLab, Bioengineering Department, Politecnico di Milano, Milano, Italy; 2 Villa Beretta, Rehabilitation Center, Valduce Hospital, Como, Italy

## Abstract

**Background:**

The restoration of walking ability is the main goal of post-stroke lower limb rehabilitation and different studies suggest that pedaling may have a positive effect on locomotion. The aim of this study was to explore the feasibility of a biofeedback pedaling treatment and its effects on cycling and walking ability in chronic stroke patients. A case series study was designed and participants were recruited based on a gait pattern classification of a population of 153 chronic stroke patients.

**Methods:**

In order to optimize participants selection, a k-means cluster analysis was performed to subgroup homogenous gait patterns in terms of gait speed and symmetry.

The training consisted of a 2-week treatment of 6 sessions. A visual biofeedback helped the subjects in maintaining a symmetrical contribution of the two legs during pedaling. Participants were assessed before, after training and at follow-up visits (one week after treatment). Outcome measures were the unbalance during a pedaling test, and the temporal, spatial, and symmetry parameters during gait analysis.

**Results and discussion:**

Three clusters, mainly differing in terms of gait speed, were identified and participants, representative of each cluster, were selected.

An intra-subject statistical analysis (ANOVA) showed that all patients significantly decreased the pedaling unbalance after treatment and maintained significant improvements with respect to baseline at follow-up. The 2-week treatment induced some modifications in the gait pattern of two patients: one, the most impaired, significantly improved mean velocity and increased gait symmetry; the other one reduced significantly the over-compensation of the healthy limb. No benefits were produced in the gait of the last subject who maintained her slow but almost symmetrical pattern. Thus, this study might suggest that the treatment can be beneficial for patients having a very asymmetrical and inefficient gait and for those that overuse the healthy leg.

**Conclusion:**

The results demonstrated that the treatment is feasible and it might be effective in translating progresses from pedaling to locomotion. If these results are confirmed on a larger and controlled scale, the intervention, thanks to its safety and low price, could have a significant impact as a home- rehabilitation treatment for chronic stroke patients.

## Background

Stroke is the leading cause of acquired adult disability [1,2]. The most common and widely recognized deficit caused by stroke is motor impairment, which typically affects one side of the body, controlateral to the brain hemisphere where the lesion occurs. The ensuing hemiparesis foresees some degrees of motor recovery depending on the severity of the lesion and on the rehabilitative training [3]. Several studies have revealed that motor experience plays a major role in the subsequent physiological reorganization occurring in the intact tissues adjacent to the lesion [4,5]. Clinical studies on central motor neuroplasticity support the role of goal-oriented, active, repetitive movements in the training of the paretic limb to enhance motor relearning and recovery [6-8].

The recovery of walking ability is considered the most important objective of the lower limb rehabilitation of individuals after stroke [9]. However, effective interventions for gait training are limited because extensive assistance is required for individuals with unstable balance, muscle weakness, and a persistent deficit in movement coordination.

In the last decade different studies suggested that significant improvements in the lower extremity function might result from using cycling as a rehabilitative method and that repetitive bilateral training provided by pedaling may have a positive effect on walking ability [10-13]. Cycling and walking share a similar kinematic pattern: both tasks are cyclical, require reciprocal flexion and extension movements of hip, knee, and ankle, and have an alternating activation of agonist/antagonist muscles in a well-timed and coordinated manner [14,15]. Furthermore, cycling avoids problems of balance and can be safely performed even from a wheelchair, without requiring expensive robotic devices or the constant supervision of a therapist which are, on the contrary, necessary to support body weight and to prevent falls during gait training. For all these reasons, leg cycling training is a safer and more economic intervention to supplement functional ambulation training after stroke and it is also becoming an interesting option for home rehabilitation of hemiparetic patients.

Providing an online feedback about patients' performance to the training improves patients' motivation, allows the therapists to assess the exercise and may lead to an enhancement in the motor relearning process [16]. This rehabilitative method is well known with the term of biofeedback (BF) and consists of the use of instrumentation to make covert physiological processes more overt. BF refers to an artificial feedback on biological quantities, transferred to a biological system (human) [17]. The use of BF re-endows patients with sensorimotor impairments with the ability to assess physiological responses and possibly to relearn self-control of those responses [18]. Besides, continued training could establish new sensory engrams and help the patients to perform tasks without feedback [19]. To maximize the effect of BF it may be important to apply it within task-oriented activity and with a feedback mode that facilitates motor relearning [18]. During pedaling, visual BF methods were developed based on EMG activity [20] and power output produced during a treatment of cycling induced by electrical stimulation [21].

Because of the laterality of the motor impairment, the postural imbalance or asymmetrical movements between the two lower limbs are commonly observed in hemiparetic patients, making the recovery of a symmetrical involvement of the two legs strictly correlated with the improvement of overground locomotion [22,23]. To minimize gait asymmetry could be clinically crucial since it may be associated with a number of negative consequences such as inefficiency, challenges to balance control, risks of musculoskeletal injury to the non-paretic lower limb and loss of bone density in the paretic lower limb [24]. During cycling, since the two legs are simultaneously acting on a single crank, not optimal solutions could be adopted by stroke patients: for example, the non- paretic leg can completely compensate for the paretic one [11], making the pedaling strategy effective in terms of speed and total power output, but strongly unbalanced. This solution could limit the possible benefits and even worsen the gait performance in terms of symmetry. To solve this problem, it could be useful to display a feedback that provides information about the forces produced at the pedals, asking patients to increase the task symmetry.

Commercial available cycle-ergometers are usually equipped with a torque sensor measuring the total torque provided by both legs at the crank, but this signal does not allow to distinguish the contribution provided by each leg during pedaling. To overcome this limitation, in our laboratory a cycle-ergometer was instrumented by mounting strain gauges on each crank arm to measure independently the torque produced by each leg during pedaling [25]. Starting from this setup, an information fusion algorithm was implemented in order to visually display to the patient an intuitive index strictly correlated with the symmetrical involvement of the two legs in terms of torques provided at the crank arms during pedaling. The aim of the present study was to develop a BF controller and to evaluate its feasibility and clinical efficacy as a rehabilitation treatment for chronic stroke patients. The hypothesis was that a 2-week BF cycling treatment might induce some improvements not only in the pedaling performance but also in the walking ability both in terms of gait speed and symmetry indices. A case series study was designed and participants were recruited based on a gait pattern classification of a population of 153 chronic stroke patients. In particular, subjects representative of each category were included in the study in order to identify those patients who can benefits the most from the proposed treatment.

## Methods

### Participants

#### Gait pattern categorization of chronic stroke patients

A population of 153 chronic stroke patients, included in a previous study [26], was chosen to perform the gait pattern categorization. All these patients underwent orthopedic procedures to correct equinovarus foot deformity and performed either prior and postoperative gait evaluation. Participants included in that study [26] satisfied the following inclusion criteria: (1) left or right hemiparesis because of ischemic or hemorrhagic stroke (diagnosis confirmed by computed tomographic scan/magnetic resonance imaging or clinical documentation or both); (2) age > 18 years; (3) time since stroke of at least 12 months; (4) mild spasticity level for all lower limb muscles (Modified Ashworth Scale ≤ 2).

The results of the postoperative gait evaluations were chosen for the gait categorization, being well representative of the walking ability of chronic stroke patients in a stable condition. During these assessments, all patients were ambulant, without using any special orthosis; some of them were helped by walking aids such as sticks (n = 70), tripods (n = 8), quadripods (n = 11), whereas the remaining group of patients (n = 64) did not use any aid.

The gait classification was based on temporal and spatial parameters able to identify the overall locomotor performance and the movement symmetry. The mean velocity was included as a variable for the cluster analysis, being defined as a reliable marker of functional disability [9] and being reported as the strongest determinant of group placement in a cluster analysis of stroke patients [27]. Besides, temporal parameters able to discriminate gait pattern in term of symmetry were chosen [24]. In particular, we considered the ratio between the values obtained by the paretic and healthy leg for the following parameters: stance time in percentage of stride time, swing time in percentage of stride time, and the intra-limb ratio of swing time against stance time. The double support time ratio was not considered in the gait categorization because it was unable to identify asymmetric individuals and the mean value did not differ a lot from healthy subjects [24].

A k-means cluster analysis was used to subgroup homogeneous gait patterns. A Mahanalobis distance criterion was adopted to eliminate any outlier from the data sample. The clustering technique is very sensitive to variables which are highly correlated, so all the variables were assessed for correlation and those highly correlated to others were removed. The selected variables were standardized before entering the cluster analysis. The Squared Euclidean distance measure was used and the number of clusters was optimized performing an a posteriori measurement of the silhouette coefficient which evaluated both cohesion and separation of the obtained centroids [28].

#### Choice of stroke participants

After having performed the cluster analysis of the population of chronic stroke patients, we chose a number of participants equal to the number of identified clusters: each patient was considered as representative of one cluster at baseline. Therefore, participants recruited in this study satisfied the same inclusion criteria of the population chosen for the gait categorization. In addition, patients were characterized by a joint mobility ranges which did not preclude pedaling (knee extension up to 150° and hip flexion up to 80°). The only exclusion criteria was an insufficient cognitive capacity to participate in the program, including receptive aphasia.

The chosen patients were prevented to perform any other lower limb intervention during the BF training.

#### Healthy subjects participants

A group of 12 healthy subjects (age 22.6 ± 3.3 years, height 171.8 cm ± 9.7 cm, weight 63.3 kg ± 8.9 kg) participated in the study in order to compute the normality ranges for both the pedaling and the walking test used to evaluate the motor recovery induced by the training.

### Experimental setup

The THERA-live™ (Medica Medizintechnik GmbH, Germany) motorized cycle-ergometer was chosen for the treatment. It was equipped with a shaft encoder for the acquisition of the crank angle and with strain gauges attached on the crank arms to measure the torque produced by each leg during pedaling [25]. During the treatment, patients sat on a chair or a wheelchair in front of the ergometer and their legs were stabilized by calf supports fixed to the pedals.

**Figure 1:**
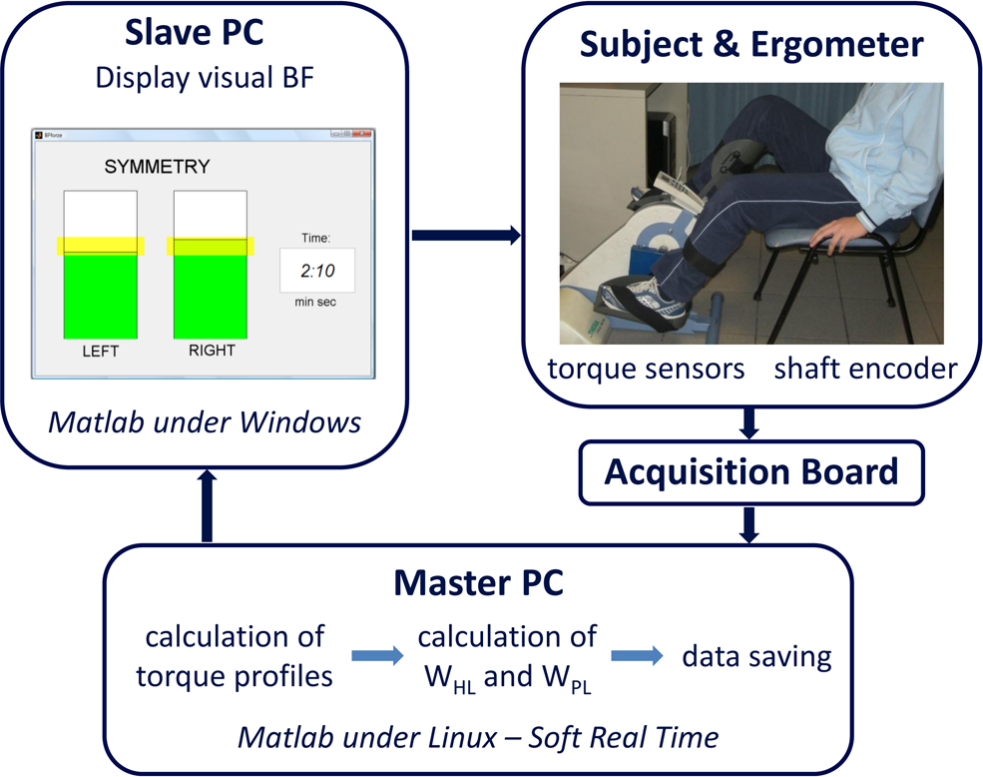
**Experimental setup used for the intervention**.

A master computer, called master PC, running Matlab/Simulink^® ^under Linux, acquired all signals coming from the ergometer with a sampling frequency of 200 Hz and calculated, at the end of each revolution, the BF indices. Then, these indices were sent to a second PC, called slave PC, which provided the visual biofeedback to the patients, displaying the values of the BF indices through a graphical interface implemented in Matlab. The communication between the PCs was obtained through LAN connection according to the UDP/IP protocol. The experimental setup is shown in Figure 1.

### Intervention

The BF treatment was performed 3 days a week for two weeks, obtaining a total of 6 sessions. Each session lasted 14 minutes:

• 1 minute of passive cycling;

• 2 minutes of voluntary cycling without visual biofeedback (VOL1);

• 8 minutes of voluntary cycling with visual biofeedback (BF phase);

• 1 minute of passive cycling;

• 2 minutes of voluntary cycling without visual biofeedback (VOL2).

Passive cycling was guaranteed by the ergometer's motor which maintained the speed at a constant value of 30 rpm.

The communication between the two PCs, shown in Figure 1, was active only during the BF phase. During the other phases the data were only acquired and saved by the master PC.

To compute the BF indices during the BF phase, the active torque profiles for each leg as function of the crank angle were obtained by subtracting the mean torque computed during passive cycling from the torque profile calculated during each revolution of voluntary pedaling. In this way, the inertial and gravitational contribution of the limbs were eliminated. Then, the BF indices for each revolution consisted of the mechanical work produced by the paretic (W_PL_) and healthy leg (W_HL_) and were computed as follows:

(1) $$W_{PL}=\int_{0^{\circ }}^{360^{\circ }}T_{PL}\left(\theta \right)d\theta$$

(2) $$W_{HL}=\int_{0^{\circ }}^{360^{\circ }}T_{HL}\left(\theta \right)d\theta$$

where *T_PL _*and *T_HL _*are the active torque profiles produced by the paretic and healthy leg, respectively, while *θ *represents the crank angle.

The slave PC displayed in real-time, at the end of each revolution, the values of work produced by the two legs, through a graphical interface consisting of two bars with a height proportional to the work values and a yellow band indicating the target (see Figure 1). Patients were asked to voluntary compensate a potential unbalance producing with each leg a value of work within the target band (yellow bands on the two bars). When the two work values were both within the yellow bands, the bars became green; otherwise they were red. To make the exercise more challenging, the target band increased the value of required work when the subjects were able to fulfill the goal for at least 7 over 10 consecutive revolutions. If the patients failed to maintain the increased target for 1 minute, the target decreased again not to discourage the subjects. The target value was subject-dependent and was fixed before the beginning of each session by means of a preliminary test. This test consisted of a 30-second period of passive cycling and a 30-second period of voluntary cycling during which patients were asked to pedal with maximal effort. At the end of the test, the values of W_PL _and W_HL _for each revolution were computed and the maximal value achieved by the paretic leg (W_PLmax_) was used to set the target interval used during the BF phase: the target could range between 80% W_PLmax _and 120% W_PLmax _and the target band was fixed at ± 10% W_PLmax_.

The proposed protocol was approved by the Ethical Committee of the rehabilitation center and each participant signed an informed consent.

### Assessment

Participants were tested before, after the intervention and in a follow-up assessment one week after the end of the treatment by means of the following assessment tests:

1. a *pedaling test*, which comprised a 1-minute period of passive cycling and a 2-minute period of voluntary cycling. The same ergometer used for the BF treatment was employed for this test. Thus, the crank angle and the torque produced independently by the paretic and healthy leg were measured and sampled at 200 Hz.

2. a *walking test *on a 10-meter walkway. Patients were asked to walk without the shoes at a self selected speed. No constraints were imposed to the subjects and neither assistive devices were used during the test. Three-dimensional kinematics of the subject's lower limbs were recorded with the Elite clinic™ (BTS, Milano, Italy) motion analysis system (8 cameras, sample rate 100 Hz) using the SAFLo protocol [29]. Ground reaction forces were measured with two dynamometric force platforms (Kistler, Winterthur, Switzerland).

### Data analysis

#### Intervention

The performance achieved daily during the BF phase was evaluated by means of the ratio between the number of symmetrical revolutions and the total number of revolutions (BF_perf_).

During VOL1 and VOL2, the values of W_PL _and W_HL _were computed for each revolution as in equations (1, 2). Then the pedaling unbalance (U) was defined as:

(3) $$U=\frac{\left|W_{HL}-W_{PL}\right|}{\left(\left|W_{HL}|+|W_{PL}\right|\right)}$$

U could range from 0 (two identical works) to 100% (WPL negative or equal to zero).

#### Assessment

The pedaling test was evaluated in terms of WHL, WPL, and U computed at each revolution. During each assessment test, considering that patients were pedaling at 30 rpm for 2 minutes, the number of revolution was about 60.

Regarding the walking test, all raw data were filtered with a fifth order causal Butterworth filter (cutoff frequency of 5 Hz) and elaborated to compute kinematics, kinetics and standard temporal and spatial gait parameters [26,29].

To evaluate gait symmetry two indices were computed:

- ST ratio, i.e., the ratio between the stance time in percentage of the stride time obtained by the paretic leg and the one obtained by the healthy leg. The ST ratio could be related to balance control issues leading the patients to shorten the paretic stance time [24].

- SV ratio, i.e., the ratio between the swing velocity obtained by the paretic leg and the one obtained by the healthy leg. The SV ratio could be related to an insufficient power generated to swing the paretic limb quickly and to an increased time for paretic foot placement [24].

All values of the temporal and spatial gait parameters reported are the mean values of 4 to 5 repeated gait trials along the walkway at the preferred speed.

#### Statistics

After having evaluated that all patients' parameters were normally distributed, an intra-subject one way Analysis of Variance (ANOVA, p < 0.05) was performed to compare pre-, post-training and follow-up outcome measurements. Moreover, a Mann-Whitney U test (p < 0.05) was used to compare patients' performance before training, after training, and at follow-up visits, with the group of healthy volunteers. A non-parametric test was preferred to identify any statistically significant difference between patients and healthy subjects, being the group of able-bodied participants not normally distributed.

## Results

### Participants

#### Gait pattern categorization

**Figure 2:**
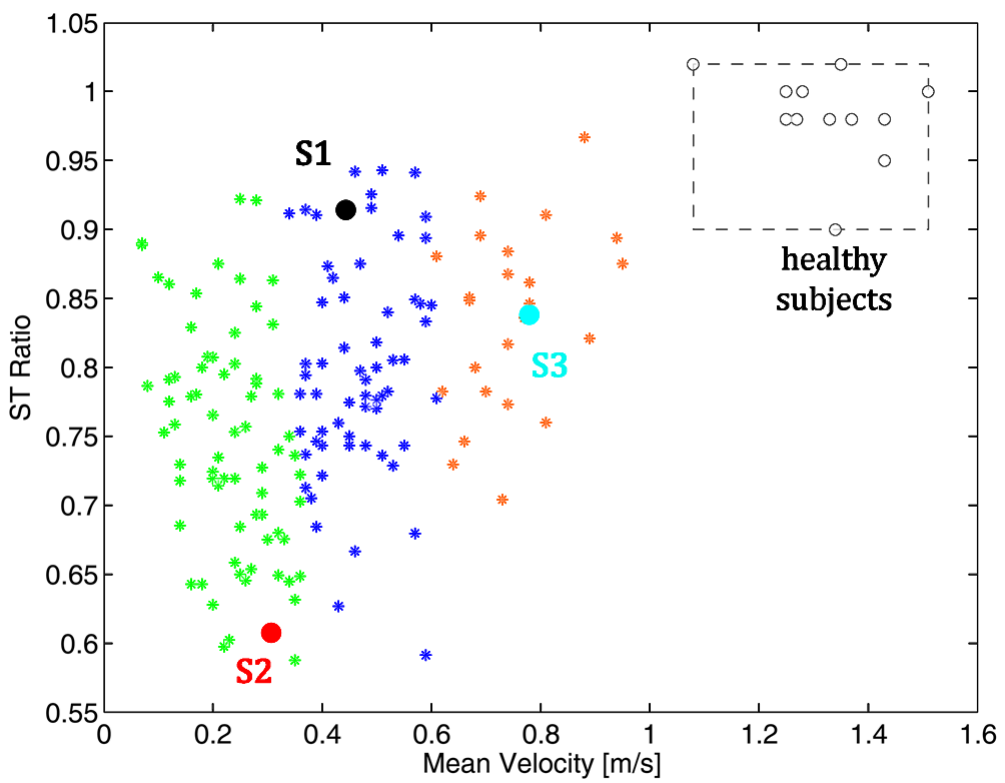
**The patients' distribution in the identified clusters at baseline**. The three clusters are reported with asterisks of different colors. S1, S2 and S3 are the black, red and light blue points, respectively. The normality ranges obtained by the group of healthy subjects are represented by the grey area (the boundary are the minimum and maximum values).

The stance time in percentage of the stride time, the swing time in percentage of the stride time, and the intra-limb ratio of the swing time against the stance time obtained in the whole population were highly correlated. This result confirmed what obtained by Patterson and collaborators [24] and, accordingly, only one of these parameters was chosen for the gait patterns categorization: the ST ratio. Thus, the two parameters used in the cluster analysis were the ST ratio and the mean velocity. Two outliers were eliminated before performing the cluster analysis. After having observed that the mean silhouette coefficient decreased moving from a three to a four-clusters solution, participants were assigned to 3 homogenous subgroups. Subgroup 1 contained 58 participants (mean ± standard deviation (SD)): ST ratio, 0.79 ± 0.08; mean velocity, 0.45 m/s ± 0.07 m/s), Subgroup 2 contained 70 participants (ST ratio, 0.75 ± 0.09; mean velocity, 0.22 m/s ± 0.07 m/s), and Subgroup 3 contained 23 participants (ST ratio 0.84 ± 0.06, mean velocity, 0.71 m/s ± 0.11 m/s). The three clusters are reported in Figure 2. The stroke population differed from the group of healthy subjects (grey area in Figure 2). This difference was more evident in terms of mean velocity than in terms of ST ratio. Indeed, some patients were characterized by an almost symmetrical gait pattern but were still significantly slower than healthy subjects. The distribution of the three clusters denotes that they were well distinct only in terms of mean velocity corroborating the hypothesis that the gait speed could be a reliable marker of function disability [9]. The population covered a huge variability of stroke patients starting from very slow walkers to quite fast patients: the minimum mean velocity was lower than 0.1 m/s, corresponding to patients who need long term care, while the maximum speed was 0.9 m/s, a value that permits unrestricted walking in the community. 

#### Patients chosen for the intervention

After giving their informed consent, 3 chronic stroke subjects, were included in the case series study. Patients' details are reported in Table 1. Two of the three participants (S2 and S3) underwent orthopedic procedures to correct equinovarus foot deformity, whereas the last one (S1) did not. Figure 2 shows the participants distribution with respect to the identified clusters before the beginning of the intervention. The selected patients were chosen in order to differ significantly from each other not only in terms of mean velocity (as it was because they belong to the three different clusters) but also in terms of gait symmetry, i.e., ST ratio. In particular, S2 was characterized by a slow gait speed and an asymmetrical gait pattern; S1 had a more symmetrical but still slow gait; S3 walked faster but his pattern was unbalanced. The treatment is mainly focused on the recovery of a symmetrical use of the legs during pedaling involving maximally the paretic one. Thus, given the significant difference between the three chosen patients, our hypothesis was that the treatment could induce a different effect in the three patients: we were expecting an increase of strength and symmetry in S2 resulting in a faster and more symmetric gait, only a decrease of asymmetry in S3, and a muscle strengthen probably resulting in a faster gait in S1.

**Table 1:** Participants baseline details

Subject	Age (years)	Gender	Etiology	Time since stroke (years)	Affected Side	Modified Ashworth Scale (0-4)	Mean Velocity (m/s) *	ST ratio (0-1) *
S1	23	female	Ischemic stroke	1	left	1	0.44 (0.03)	0.92 (0.04)
S2	51	male	Ischemic stroke	10	right	1	0.31 (0.04)	0.57 (0.05)
S3	27	male	Hemorrhagic stroke	9	right	2	0.78 (0.04)	0.80 (0.04)

* Values: Mean (SD)

### Normality Ranges

In the pedaling test, the healthy subject group obtained a median value of unbalance equal to 1.50% with an interquartile range (IQR) of 3.05%.

The normality ranges obtained during the walking test in terms of spatio-temporal variables and symmetry parameters are reported in Table 2.

**Table 2:** Normality ranges for the walking assessment test

	Leg	Median (IQR)
Stance Time [%stride]	Right Left	59 (1) 58 (2)
Swing Time [%stride]	Right Left	40 (1) 41 (2)
Stride Time [ms]	Right Left	1045 (112) 1065 (100)
Stride Length [mm]	Right Left	1374 (140) 1393 (159)
Swing Velocity [m/s]	Right Left	3.27 (0.20) 3.17 (0.27)
Mean Velocity [m/s]		1.33 (0.12)
ST Ratio		0.98 (0.02)
SV Ratio		0.97 (0.03)

Values: Median (IQR) of the spatio-temporal and symmetry parameters computed on the healthy subjects group during the walking test.

### Intervention

**Figure 3:**
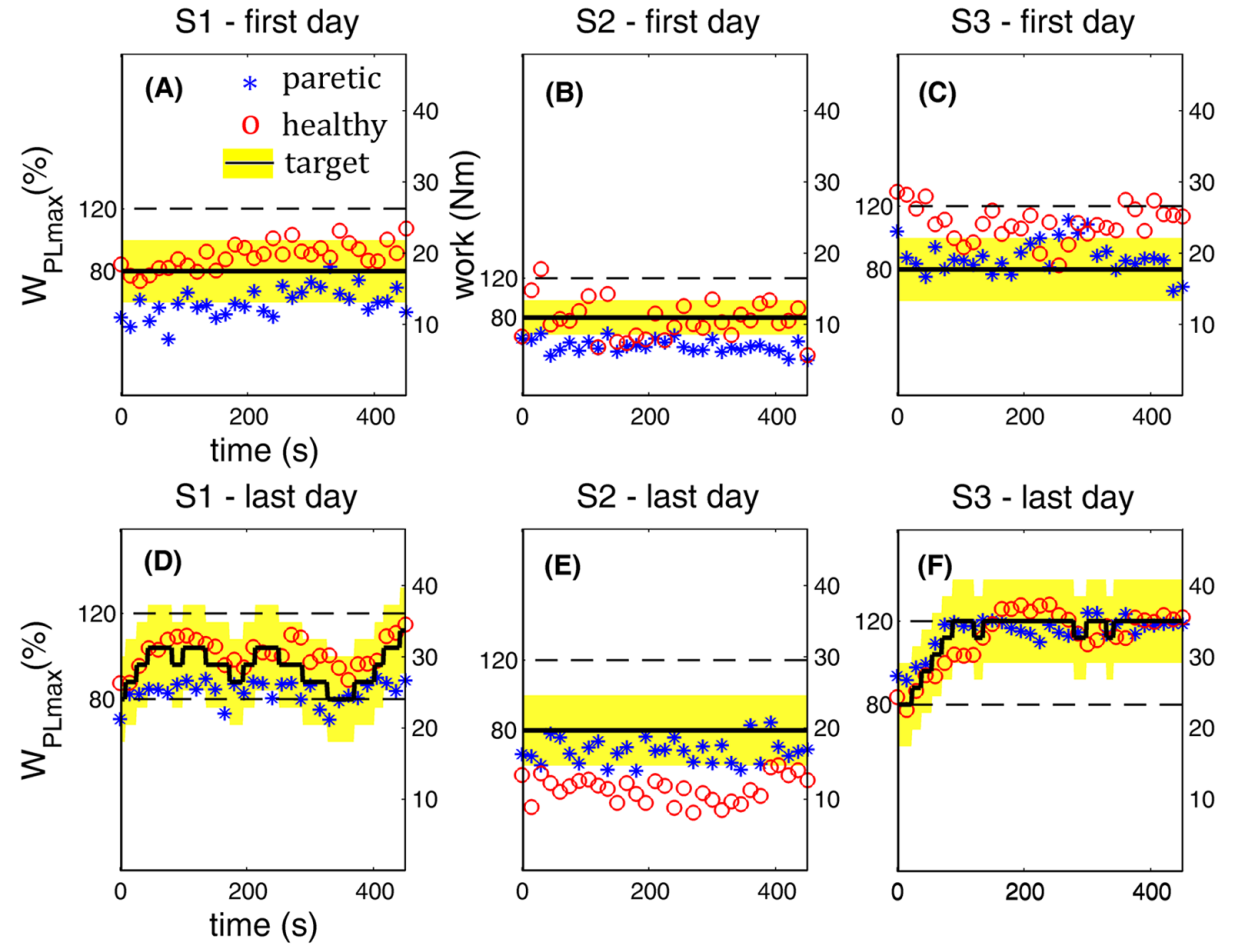
**Performance obtained during the BF phase in the first and last day of treatment**. Results obtained by the three patients in the first (upper panels) and last (lower panels) day of treatment during the BF phase. Each asterisk and circle indicate the mean value, among 10 consecutive revolutions, of the work produced by the paretic and healthy leg, respectively. The black line shows the target value and the surrounding yellow area represents the tolerance band. In all panels, double vertical axes are used to indicate the absolute work value and the minimum and maximum target values in percentage of W_PLmax_.

Figure 3 depicts a comparison between the performance obtained by the three patients during the first (upper panels) and the last (lower panels) day of treatment in terms of work produced by the two legs during the 8 minutes of voluntary cycling with visual biofeedback (BF phase).

In the first day of treatment, S1 (panel (A)) was not able to produce a symmetric pedaling. Indeed, the work values produced by the paretic and non-paretic leg (asterisks and circles, respectively) were not included in the tolerance area (yellow band). It is noticeable that her performance improved after treatment: in the last day (panel (D)), she was also able to achieve a symmetric pedaling, and, thus, the target value of work (black line) increased. This symmetric pedaling was only partly maintained in the middle part of the session (sometimes the target decreased because she was tired or not able to be concentrated for a long time), but then, in the final part, she was able to reach the maximal level of the target (120% WPLmax). Furthermore, the target work used in the last day of treatment (ranges from 25 Nm to 35 Nm) was higher than the one used in the first day (about 18 Nm). This result suggested us that S1 was able to understand and exploit properly the visual biofeedback.

S2 was able to achieve a symmetric pedaling neither in the first nor in the last day of treatment (panels (B) and (E)). However, in the last day of treatment, he reversed his pedaling strategy: he was very concentrated on pedaling with the paretic side, trying to relax the healthy one. Thus, his pedaling resulted to be unbalanced in favor of the paretic side. In particular, the target value and the work produced by the paretic leg during the last day of treatment were doubled with respect to the values produced during the first day, implying an increase of strength achieved by S2.

Finally, S3 was overusing the healthy leg in the first day of treatment (panel (C)), while he succeeded in understanding the visual biofeedback in the last day of treatment. Indeed, he achieved and maintained a symmetric pedaling (panel F): the target work increased till the maximal value and was maintained for the whole period of the BF phase. In addition, the treatment induced an increase of force also in S3, being the target work used in the last day of treatment about the double of the one used in the first day.

**Figure 4:**
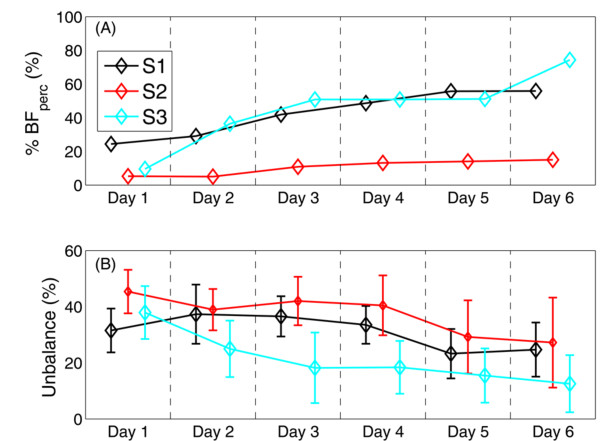
**Day-by-day performance during the intervention**. Trend of the performance obtained during the 6 days of treatment in terms of BF_perf_, computed during the BF phase (panel (A)), and unbalance (panel (B)) during VOL1.

Figure 4 shows the performance obtained daily by the three patients. All patients were able to increase their performance (BF_perf_ in panel (A)) during the treatment, implying the efficacy and easiness of the visual feedback given to the patients. Furthermore, the unbalance computed during VOL1 decreased over time for all patients, suggesting that they learnt how to execute a symmetrical task (panel (B)), also without being helped by the feedback.

### Assessment

Table 3 reports the mean and the standard deviation values of the works produced by the paretic and healthy legs, and of the pedaling unbalance obtained in the pre, post-treatment, and follow-up assessment, while Table 4 reports the results obtained during the walking assessment test by the three participants. In what follows, the results are presented case by case.

**Table 3:** Results of the pedaling assessment test

		PRE	POST	FU	P *	P * (pre vs post)	P * (pre vs fu)	P * (post vs fu)
**S1**								
	U (%)	31.5 (8.0)	24.7 (9.6)	18.3 (7.3)	*< 0.01*	*< 0.01*	*< 0.01*	*< 0.01*
	W_HL _(Nm)	47.8 (5.5)	45.0 (5.8)	43.3 (5.6)	*< 0.01*	*< 0.01*	*< 0.01*	0.07
	W_PL _(Nm)	25.2 (5.5)	27.4 (5.3)	30.1 (5.6)	*< 0.01*	*0.01*	*< 0.01*	*0.01*
**S2**								
	U (%)	45.4 (7.8)	29.2 (13.0)	39.9 (13.7)	*< 0.01*	*< 0.01*	*0.02*	*< 0.01*
	W_HL _(Nm)	35.0 (6.5)	43.5 (12.7)	43.1 (10.3)	*< 0.01*	*< 0.01*	*< 0.01*	0.97
	W_PL _(Nm)	13.0 (2.6)	25.7 (10.9)	19.3 (7.9)	*< 0.01*	*< 0.01*	*< 0.01*	*< 0.01*
**S3**								
	U (%)	38.1 (9.4)	12.4 (10.1)	13.6 (10.6)	*< 0.01*	*< 0.01*	*< 0.01*	0.69
	W_HL _(Nm)	78.5 (8.3)	36.2 (4.3)	42.8 (3.9)	*< 0.01*	*< 0.01*	*< 0.01*	*< 0.01*
	W_PL _(Nm)	35.9 (9.2)	29.3 (4.9)	33.7 (6.8)	*< 0.01*	*< 0.01*	0.06	*< 0.01*

Values: Mean (SD)

* P: Significance level of one way ANOVA (p < 0.05)- Post-hoc: Scheffè

U indicates the pedaling unbalance; W_HL _and W_PL_, the works produced by the healthy and paretic legs, respectively.

**Table 4:** Results of walking assessment test

	Leg	PRE	POST	FU	P *	P * (pre vs post)	P * (pre vs fu)	P * (post vs fu)
**S1**								
Stance Time	P	64 (2)	63 (2)	65 (3)	0.43			
[%stride]	H	70 (2)	72 (2)	71 (3)	0.48			
Swing Time	P	36 (2)	37 (2)	35 (3)	0.43			
[%stride]	H	30 (2)	28 (2)	29 (3)	0.48			
Stride Time	P	1896(121)	1754 (38)	1764(129)	0.10			
[ms]	H	1880 (84)	1742(100)	1770(125)	0.13			
Stride Length	P	859 (18)	817 (26)	845 (34)	0.29			
[mm]	H	820 (25)	812 (51)	872 (40)	0.07			
Swing Velocity	P	1.27(0.08)	1.28(0.04)	1.40(0.14)	0.11			
[m/s]	H	1.47(0.05)	1.67(0.15)	1.69(0.13)	*0.03*	0.07	*0.04*	0.95
Mean Velocity [m/s]		0.44(0.03)	0.47(0.01)	0.49(0.03)	0.07			
ST Ratio		0.92(0.04)	0.89(0.03)	0.92(0.04)	0.32			
SV Ratio		0.86(0.05)	0.77(0.09)	0.83(0.11)	0.30			
**S2**								
Stance Time	P	48 (4)	54 (2)	53 (2)	*0.03*	*0.04*	*0.02*	0.76
[%stride]	H	79 (8)	69 (1)	66 (3)	*0.01*	*0.04*	0.10	0.97
Swing Time	P	52 (4)	46 (2)	47 (2)	*0.03*	*0.04*	*0.02*	0.76
[%stride]	H	21 (8)	31 (1)	34 (3)	*0.01*	*0.04*	0.10	0.97
Stride Time	P	1870(206)	1400 (96)	1663 (93)	*< 0.01*	*< 0.01*	0.96	*< 0.01*
[ms]	H	2402(515)	1528(101)	1630 (79)	*< 0.01*	*< 0.01*	*0.03*	0.90
Stride Length	P	637 (46)	745 (31)	630 (13)	*< 0.01*	*< 0.01*	0.16	*< 0.01*
[mm]	H	619 (72)	788 (37)	666 (20)	*< 0.01*	*< 0.01*	0.50	*0.03*
Swing Velocity	P	0.66(0.27)	1.12(0.07)	0.81(0.07)	*< 0.01*	*< 0.01*	0.16	*< 0.01*
[m/s]	H	1.37(0.25)	1.66(0.14)	1.21(0.07)	*0.02*	*0.05*	0.50	*0.02*
Mean Velocity [m/s]		0.31(0.04)	0.5 (0.03)	0.40(0.01)	*< 0.01*	*< 0.01*	*0.04*	*< 0.01*
ST Ratio		0.57(0.05)	0.72(0.03)	0.83(0.05)	*< 0.01*	*< 0.01*	*< 0.01*	*0.02*
SV Ratio		0.53(0.14)	0.70(0.06)	0.67(0.03)	*0.02*	*0.05*	0.20	0.92
**S3**								
Stance Time	P	57 (2)	56 (2)	55 (3)	0.38			
[%stride]	H	68 (3)	65 (1)	65 (2)	*0.04*	*0.05*	0.14	0.91
Swing Time	P	43 (2)	44 (2)	45 (3)	0.38			
[%stride]	H	32 (3)	35 (1)	35 (2)	*0.04*	*0.05*	0.14	0.91
Stride Time	P	1264 (52)	1333 (84)	1297 (24)	0.24			
[ms]	H	1324 (55)	1332 (68)	1357 (77)	0.76			
Stride Length	P	986 (30)	1016 (59)	1012 (87)	0.42			
[mm]	H	1026 (19)	1053 (50)	1088 (69)	0.20			
Swing Velocity	P	1.82(0.14)	1.74(0.13)	1.73(0.05)	0.53			
[m/s]	H	2.45(0.27)	2.25(0.16)	2.31(0.26)	0.38			
Mean Velocity [m/s]		0.78(0.04)	0.78(0.06)	0.78(0.04)	0.93			
ST Ratio		0.80(0.04)	0.87(0.05)	0.81(0.07)	0.15			
SV Ratio		0.75(0.09)	0.78(0.07)	0.76(0.09)	0.79			

Values: Mean (SD)

* P: Significance level of one way ANOVA (p < 0.05) - Post-hoc: Scheffè

P indicates the paretic side; H, the healthy one.

#### S1

After the 2-week treatment, S1 achieved a significant decrease of the unbalance (Table 3) obtained by a slight increase of W_PL _and a slight decrease of W_HL_. The pedaling unbalance was further reduced in the follow-up assessment. Although the treatment induced a significant improvement of the pedaling unbalance, the U-test performed to compare the performance of S1 with the group of healthy subjects (median [IQR]: unbalance,1.50% [3.05%]) showed significant differences at all assessment tests (pre-, post-training and follow-up).

The results obtained in the pedaling assessment tests were not translated to improvements in terms of walking ability. Indeed, S1 at baseline was characterized by a slow and almost symmetric gait and the treatment did not induce any gait improvement in her locomotor performance (Table 4). The only significant variation in the gait parameters was an increase of the swing velocity of the healthy leg but it seems not to be related to the treatment because the post-hoc analysis revealed that a difference existed between the pre-treatment and the follow-up assessment but did not soon after the end of the training.

The U-test performed to compare each walking assessment of S1 with the group of healthy subjects showed that S1 resulted not significantly different from the healthy subject group in terms of ST ratio and SV ratio during the pre-training and the follow-up assessment.

#### S2

S2 significantly improved his pedaling unbalance after treatment. To achieve this performance, he increased both values of work, but WPL increased the more (it was doubled after treatment with respect to baseline). Comparing the follow-up with the post-training assessment, S2 worsened the unbalance, although his pedaling remained significantly more symmetrical than in the pre-treatment evaluation. The pedaling unbalance was always very different from the healthy subject normality range (U-test, p < 0.01).

The BF treatment seemed to be beneficial in terms of walking ability recovery for S2 (Table 4). Indeed, the treatment produced a statistically reliable increase of the mean velocity, due to both a significant increase of the stride length and a significant decrease of the stride time for the two legs. These improvements were maintained at follow-up keeping the mean velocity significantly higher than in the pre-training assessment, even if it was lower than at post-treatment evaluation. Furthermore, S2 changed his gait pattern: he modified the step temporization producing a more symmetrical balance between the stance and swing phases, and maintained this temporization in the follow-up assessment. The post treatment evaluation was also characterized by an increase of the swing velocity of the paretic leg (p < 0.01); this latter benefit was not maintained at follow-up. All the gait parameters obtained by S2 were always outside the normality ranges.

#### S3

In the pedaling assessment test carried out after treatment, S3 decreased significantly his unbalance with respect to the pre-test. This improvement was produced by a significant reduction of the performance of both legs, particularly the healthy one. The results obtained were maintained at follow-up. In all the pedaling assessment tests, S3 produced a significant difference with respect to the healthy subject group in terms of unbalance.

The BF treatment induced a significant change in terms of gait pattern timing also for S3: the stance and swing time percentages with respect to the stride time significantly changed in the healthy leg (p = 0.04). This adaptation of the healthy leg behavior corresponded to a slightly longer stride length for both paretic and healthy side. All these progresses were preserved at follow-up.

Among all spatio-temporal parameters reported in Table 4, only the stance and swing time of the paretic leg of S3 resulted to be always included in the normality ranges.

**Figure 5:**
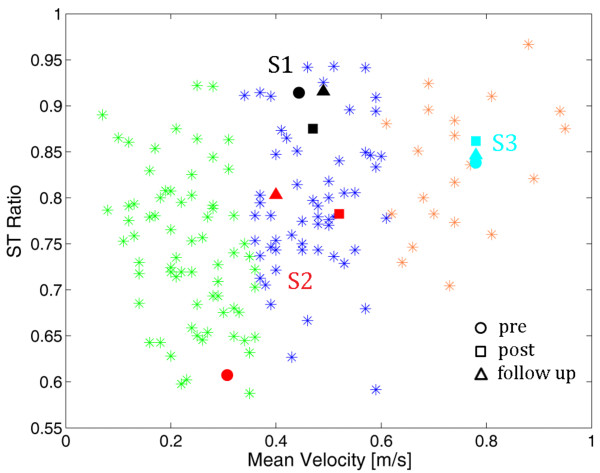
**The patients' distribution in the identified clusters**. S1, S2 and S3 are the black, red and light blue points, respectively. The pre-training, post-training and follow-up assessments are reported with a circle, square and triangle, respectively.

Figure 5 shows the distribution of the 3 patients with respect to the gait pattern categorization in the three gait assessments. The gait performance obtained by S2 was so improved after treatment that he could change his cluster. The new class was maintained at follow-up.

## Discussion

The present work investigated the feasibility and utility of a biofeedback cycling treatment and its effects on cycling unbalance and walking parameters in three case studies of chronic stroke patients. After having performed a gait pattern categorization of a population of 153 chronic stroke patients, three participants, each of them representative of one of the clusters in which the population resulted to be divided, were enrolled in the study: S1 presented a slow and almost symmetric gait; S2, the most impaired one, was characterized by a very asymmetrical and slow gait; S3 showed the strongest and fastest gait, but still exhibited gait asymmetry due to an overuse of the healthy limb. In our experimental approach we tried to keep to the key ingredients for motor functional recovery providing an intensive and repetitive task training able to maintain a high active involvement of the patients during the intervention [18]. Furthermore, to maximize patients' involvement we increased the task difficulty as participants' skill improved.

The results obtained by the three patients emphasized the importance of developing biofeedback treatment approaches that are effective in maximizing underlying mechanisms responsible for neurological and adaptive recovery in individuals with hemiparesis, even in chronic state. The chosen visual feedback resulted simple to be understood by patients. Indeed, as shown in Figure 4, all patients improved their performance obtained during the biofeedback phase.

The most appealing questions for the proposed treatment are whether the effects obtained during the BF phase can be maintained also when the exercise is performed without the visual BF and most of all whether the effects obtained on cycling symmetry can be translated to the walking performance. To reply to the first question, it can be noticed that in the post-treatment assessments all patients significantly reduced their pedaling unbalance with respect to baseline (Table 3). Concerning the second question, the treatment, even short, seems to produce some modifications on the gait kinematic pattern in two of the three chronic patients.

The most significant improvement was obtained by S2, the patient who strengthened the most the paretic side (he doubled W_PL _after training, see Table 3). This subject was characterized by a very slow and asymmetrical gait at baseline (mean velocity = 0.32 m/s; ST ratio = 0.57 ± 0.05; SV ratio 0.53 ± 0.14). After treatment he doubled the swing velocity of the paretic leg, meaning that the patient started to produce the inertia to generate the step also with the paretic leg and started to translate this inertia in distal propulsive force improving the kinetic both at the foot and knee (results not shown). This result is confirmed by a significant improvement of gait symmetry that was also maintained (SV ratio) or further increased (ST ratio) at follow-up. In the post-treatment assessment, S2 obtained also a significant increase of the mean gait speed that is a crucial functional indicator of an improved walking ability [30]. The self-selected speed of S2 increased of the 67% with respect to baseline changing from 0.32 m/s to 0.51 m/s; this increase can be recognized as enough to change from a category of a household walker to a full community walker, and thus it can indicate a potential improvement in the quality of life following the proposed treatment [31].

The progress on walking velocity was preserved at follow-up even if at lower levels, implying that a 2-week treatment was probably not enough to induce permanent changes in the walking ability of S2.

The biofeedback cycling treatment produced some benefits also in the gait pattern of S3. He obtained a significant change in the alternation of stance and swing phases of the healthy limb, thus reducing the compensation strategy used during walking. This behavior was exactly the same he adopted also to reduce unbalance during cycling; indeed, he significantly reduced the work produced by the healthy limb. For this patient, the improvements induced by the treatment were maintained one week after the end of the intervention. The significant benefits obtained in pedaling unbalance were not translated in a significant improvement in gait symmetry even if a slight increase of both SV and ST ratio were obtained after treatment (see S3 results in Table 4).

Finally, S1 did not change her gait pattern after only 2 weeks of treatment and maintained her slow but safe and almost symmetrical gait mostly due to a general hypotonia. Thus, our hypothesis that such a BF treatment could induce a general strengthen in S1 resulting in an increase of her gait speed was not valid. Our preliminary results seem to suggest that the proposed treatment was not useful for a patient characterized by gait symmetry parameters included in the normality ranges before treatment.

The main limitation of this study is that all the presented results need to be substantiated by testing on a larger number of patients. Furthermore, the results seem to suggest that the small number of sessions administered (n = 6) is not enough to have a clear idea about the training potentiality in producing the carry-over effect from pedaling to overground locomotion. In addition, our follow-up assessment, very close to the end of the treatment, is not representative of a long term effect of training but gives only a first indication about the maintenance of the induced motor recovery. At last, our study is limited to a population of chronic stroke patients with mild spasticity (Modified Ashworth Scale ≤ 2) and this does not cover the whole stroke population [32].

## Conclusions

The results of this study suggest that a treatment of only 6 days is able to produce improvements in terms of pedaling unbalance and, sometimes, also in terms of walking ability, but probably a more prolonged treatment would be more effective in translating progresses from pedaling performance into locomotor capability and in maintaining the results over time. Naturally, the duration of the treatment has to be optimized depending on the specific patients' condition in strict collaboration with physicians. This study tries also to give some suggestions about how to choose patients' categories which can avail themselves of the treatment. The treatment seems to be beneficial for people with a very asymmetrical and inefficient gait such as S2 and for people that make an overuse of the healthy leg to compensate for their asymmetry, like S3.

Certainly a more robust statistical study (e.g. randomized controlled trial) is required to provide a clear evidence that a pedaling treatment with visual biofeedback significantly improves walking ability in chronic stroke patients. To validate a carry-over effect from pedaling to overground locomotion, a more prolonged treatment (e.g. a 4-week intervention) will be tested on a targeted category of individuals with stroke, i.e., patients included in the initial cluster of S2, that are characterized by a very asymmetrical and slow gait. If the effect is demonstrated on a larger and controlled scale, the proposed intervention, thanks to its safety and low price, could really have an impact also as a home-rehabilitation treatment for chronic stroke patients.

## Abbreviations

ANOVA: analysis of variance; BF: biofeedback; EMG: electromyography; VOL1: first phase of voluntary cycling without visual biofeedback; VOL2: second phase of voluntary cycling without visual biofeedback; W_PL_: Work produced by the paretic leg; W_HL_: Work produced by the healthy leg; T_PL_: Active torque produced by the paretic leg; T_HL_: Active torque produced by the healthy leg; W_PLMAX_: maximum work value produced by the paretic leg; U: unbalance; ST ratio: the ratio between the stance time in percentage of the stride time obtained by the paretic leg and the one obtained by the healthy leg; SV ratio: the ratio between the swing velocity obtained by the paretic leg and the one obtained by the healthy leg; BF_perf_: performance obtained daily by the three patients during the BF phase; S1: Subject 1; S2: Subject 2; S3: Subject 3; SD: standard deviation; IQR: interquartile range.

## Competing interests

The authors declare that they have no competing interests.

## Authors' contributions

SF participated to study design, data collection and analysis, and manuscript writing; EA participated to study design, data collection and analysis, and manuscript definition; PR participated at data collection; EG participated at data collection; FM participated to recruitment of stroke patients and manuscript revision; GF participated to study design and manuscript revision; AP participated to study design, and manuscript revision.

All authors read and approved the final manuscript.

## References

[B1] Aminoff MJ Greenberg DA Simon RP Clinical Neurology 2005 6 McGraw- Hill/Lange

[B2] Francescutti C Mariotti S Simon G D'Errigo P Di Bidino R The impact of stroke in Italy: first step for a national burden of disease study Disability and Rehabilitation 2005 27 5 229 240 10.1080/09638280400006457 16025750

[B3] Rossini PM Calautti C Pauri F Baron JC Post stroke plastic re-organization in adult brain Lancet Neurol 2003 2 8 493 502 10.1016/S1474-4422(03)00485-X 12878437

[B4] Nelles G Spiekermann G Jueptner M Leonhardt G Muller S Gerhard H Diener C Reorganization of sensory and motor system in hemiplegic stroke patients: a positron emission tomography study Stroke 1999 30 1510 6 10.1161/01.STR.30.8.1510 10436092

[B5] Cao Y D'Olhaberriague L Vikingstad EM Levine SR Welch KM Pilot study of functional MRI to assess cerebral activation of motor function after post-stroke hemiparesis Stroke 1998 29 112 22 10.1161/01.STR.29.1.112 9445338

[B6] Krakauer JW Motor learning: its relevance to stroke recovery and neurorehabilitation Curr Opin Neurol 2006 19 1 84 90 10.1097/01.wco.0000200544.29915.cc 16415682

[B7] Lotze M Braun C Birbaumer N Anders S Cohen LG Motor learning elicited by voluntary drive Brain 2003 126 4 866 872 10.1093/brain/awg079 12615644

[B8] Langhorne P Coupar F Pollock A Motor recovery after stroke: a systematic review Lancet Neurol 2009 8 8 741 54 10.1016/S1474-4422(09)70150-4 19608100

[B9] Dickstein R Rehabilitation of gait speed after stroke: a critical review of intervention approaches Neurorehabil Neural Repair 2008 22 6 649 660 10.1177/15459683080220060201 18971380

[B10] Potempa K Lopez M Braun LT Szidon JP Fogg L Tincknell T Physiological outcomes of aerobic exercise training in hemiparetic stroke patients Stroke 1995 26 1 101 105 10.1161/01.STR.26.1.101 7839377

[B11] Brown DA Kautz SA Increased workload enhances force output during pedaling exercise in persons with poststroke hemiplegia Stroke 1998 29 3 598 606 10.1161/01.STR.29.3.598 9506599

[B12] Brown DA Nagpal S Chi S Limb-load cycling program for locomotor intervention following stroke Phys Ther 2005 85 2 159 68 15679467

[B13] Tang A Sibley KM Thomas SG Bayley MT Richardson D McIlroy WE Brooks D Effects of an aerobic exercise program on aerobic capacity, spatiotemporal gait parameters, and functional capacity in subacute stroke Neurorehabil Neural Repair 2009 23 4 398 406 19088223 10.1177/1545968308326426

[B14] Raasch C Zajac F Locomotor strategy for pedaling: muscle groups and biomechanical functions J Neurophysiol 1999 81 515 525 10.1152/jn.1999.82.2.515 10444651

[B15] Ting LH Kautz SA Brown DA Zajac FE Phase reversal of biomechanical functions and muscle activity in backward pedaling J Neurophysiol 1999 81 544 551 10036258 10.1152/jn.1999.81.2.544

[B16] Sveistrup H Motor rehabilitation using virtual reality J Neuroengineering and rehabilitation 2004 10 1 10 10.1186/1743-0003-1-10 PMC546406 15679945

[B17] Riener R Lünenburger L Colombo G Human-centered robotics applied to gait training and assessment J Rehabil Res Dev 2006 43 5 679 694 10.1682/JRRD.2005.02.0046 17123208

[B18] Huang H Wolf SL He J Recent developments in biofeedback for neuromotor rehabilitation J Neuroeng Rehabil 2006 3 11 10.1186/1743-0003-3-11 16790060 PMC1550406

[B19] Wolf SL Electromyographic biofeedback applications to stroke patients, A critical review Phys Ther 1983 63 1448 1459 6351119 10.1093/ptj/63.9.1448

[B20] Brown DA DeBacher GA Bicycle ergometer and electromyographic feedback for treatment of muscle imbalance in patients with spastic hemiparesis. Suggestion from the field Phys Ther 1987 67 11 1715 1719 3671508 10.1093/ptj/67.11.1715

[B21] McRae CGA Johnston TE Lauer RT Tokay AM Lee SCK Hunt KJ Cycling for children with neuromuscular impairments using electrical stimulation-development of tricycle- based systems Med Eng Phys 2009 31 6 650 9 10.1016/j.medengphy.2008.12.005 19196537

[B22] Chen HY Chen SC Jason Chen JJ Fu LL Wang YL Kinesiological and kinematical analysis for stroke subjects with asymmetrical cycling movement patterns J Electromyogr Kinesiol 2005 15 6 587 595 10.1016/j.jelekin.2005.06.001 16051498

[B23] Ambrosini E Ferrante S Schauer T Ferrigno G Molteni F Pedrocchi A Design of a symmetry controller for cycling induced by electrical stimulation - Preliminary results on post-acute stroke patients Artificial Organs 2010 34 8 663 667 20528850 10.1111/j.1525-1594.2009.00941.x

[B24] Patterson KK Gage WH Brooks D Black SE McIlroy WE Evaluation of gait symmetry after stroke: a comparison of current methods and recommendations for standardization Gait Posture 2010 31 2 241 246 10.1016/j.gaitpost.2009.10.014 19932621

[B25] Comolli L Ferrante S Pedrocchi A Bocciolone M Ferrigno G Molteni F Metrological characterization of a cycle ergometer to optimise the cycling induced by functional electrical stimulation on patients with stroke Med Eng & Phys 2010 32 339 348 10.1016/j.medengphy.2010.01.005 20171923

[B26] Carda S Bertoni M Zerbinati P Rossini M Magoni L Molteni F Gait changes after tendon functional surgery for equinovarus foot in patients with stroke: assessment of temporo- spatial, kinetic, and kinematic parameters in 177 patients Am J Phys Med Rehabil 2009 88 4 292 301 10.1097/PHM.0b013e318198b593 19190482

[B27] Mulroy S Gronley J Weiss W Newsam C Perry J Use of cluster analysis for gait pattern classificationof patients in the early and late recovery phases following stroke Gait Posture 2003 18 114 25 10.1016/S0966-6362(02)00165-0 12855307

[B28] Rousseeuw PJ Silhouettes: a Graphical Aid to the Interpretation and Validation of Cluster Analysis Computational and Applied Mathematics 1987 20 53 65 10.1016/0377-0427(87)90125-7

[B29] Frigo C Rabuffetti M Kerrigan DC Deming LC Pedotti A Functionally oriented and clinically feasible quantitative gait analysis method Med Biol Eng Comput 1998 36 179 185 10.1007/BF02510740 9684457

[B30] Bowden MG Balasubramanian CK Behrman AL Kautz SA Validation of a speed-based classification system using quantitative measures of walking performance poststroke Neurorehabilitation and Neural Repair 2008 22 672 675 10.1177/1545968308318837 18971382 PMC2587153

[B31] Jonsdottir J Cattaneo D Recalcati M Regola A Rabuffetti M Ferrarin M Casiraghi A Task- oriented biofeedback to improve gait in individuals with chronic stroke: motor learning approach Neurorahabilitation and Neural Repair 2010 24 5 478 485 10.1177/1545968309355986 20053951

[B32] Brainin M Norrving B Sunnerhagen KS Goldstein LB Cramer SC Donnan GA Duncan PW Francisco G Good D Graham G Kissela BM Olver J Ward A Wissel J Zorowitz R International PSS Disability Study Group Poststroke chronic disease management: towards improved identification and interventions for poststroke spasticity- related complications International Journal of Stroke 2011 6 42 46 10.1111/j.1747-4949.2010.00539.x 21205240

